# Accurate Identification of Closely Related *Mycobacterium tuberculosis* Complex Species by High Resolution Tandem Mass Spectrometry

**DOI:** 10.3389/fcimb.2021.656880

**Published:** 2021-06-22

**Authors:** Amol O. Bajaj, Suraj Saraswat, Juha E. A. Knuuttila, Joanna Freeke, J. Benjamin Stielow, Adam P. Barker

**Affiliations:** ^1^ Research & Development, Associated Regional and University Pathologists, Inc. (ARUP) Institute for Clinical and Experimental Pathology, Salt Lake City, UT, United States; ^2^ Research & Development, Thermo Fisher Scientific, Helsinki-Vantaa, Finland; ^3^ Centre for Infectious Diseases, Radboud University Medical Center (UMC), Nijmegen, Netherlands; ^4^ Research & Development, Thermo Fisher Scientific, Landsmeer, Netherlands

**Keywords:** mass spectrometry, species delimitation, clinical mycobacteriology, *Mycobacterium tuberculosis*, clinical diagnostics

## Abstract

Rapid and accurate differentiation of *Mycobacterium tuberculosis* complex (*MTBC*) species from other mycobacterium is essential for appropriate therapeutic management, timely intervention for infection control and initiation of appropriate health care measures. However, routine clinical characterization methods for *Mycobacterium tuberculosis* (*Mtb*) species remain both, time consuming and labor intensive. In the present study, an innovative liquid Chromatography-Mass Spectrometry method for the identification of clinically most relevant *Mycobacterium tuberculosis* complex species is tested using a model set of mycobacterium strains. The methodology is based on protein profiling of *Mycobacterium tuberculosis* complex isolates, which are used as markers of differentiation. To test the resolving power, speed, and accuracy of the method, four ATCC type strains and 37 recent clinical isolates of closely related species were analyzed using this new approach. Using different deconvolution algorithms, we detected hundreds of individual protein masses, with a subpopulation of these functioning as species-specific markers. This assay identified 216, 260, 222, and 201 proteoforms for *M. tuberculosis* ATCC 27294™, *M. microti* ATCC 19422™, *M. africanum* ATCC 25420™, and *M. bovis* ATCC 19210™ respectively. All clinical strains were identified to the correct species with a mean of 95% accuracy. Our study successfully demonstrates applicability of this novel mass spectrometric approach to identify clinically relevant *Mycobacterium tuberculosis* complex species that are very closely related and difficult to differentiate with currently existing methods. Here, we present the first proof-of-principle study employing a fast mass spectrometry-based method to identify the clinically most prevalent species within the *Mycobacterium tuberculosis* species complex.

## Introduction

*Mycobacterium tuberculosis* is responsible for one of the most devastating and chronic infectious diseases known to science and is an important and formidable human pathogen that has claimed nearly 1.2 million lives in 2019 (Andersen and Brennan, 1994). Approximately 10 million people were infected in 2019 and the pathogen can persist in a hidden form if undiagnosed for a long period of time within the human host (Bloom and Murray, 1992; WHO, 2020). Almost 10% of an infected population will progress to disease (active tuberculosis) following a latent period (from weeks to decades) (Bloom and Murray, 1992). Multidrug-resistant tuberculosis has recently emerged (MDR-TB) and represents an enormous challenge to the public health system worldwide. Worldwide in 2019, close to half a million people developed rifampicin-resistant TB (RR-TB), of which 78% had multidrug-resistant TB (MDR-TB). The three countries with the largest share of the global burden were India (27%), China (14%) and the Russian Federation (8%) (WHO, 2020). The largest increase seen globally in the number of nontuberculosis mycobacterial (NTM) and *MTBC* infections was observed in patients who had already contracted the human immunodeficiency virus (HIV).

*Mycobacterium tuberculosis* complex encompasses a group of organisms of paramount clinical relevance that cause tuberculosis (TB) in humans and animals. TB in humans is caused mainly by *M. tuberculosis* comprising an enormous diversity of genetic lineages (e.g. including its formerly known *M. africanum*) but also by other members of *MTBC*. Zoonotic TB is caused by its less well understood animal-associated subspecies *M. bovis*, *M. canetti, M. caprae*, *M. pinnipedii*, *M. suricattae*, *M. orygis*, *M. microti*, and *M. mungi* (Gagneux and Small, 2007; Alexander et al., 2010; Gupta et al., 2018; Riojas et al., 2018; Lipworth et al., 2019). However, not all zoonotic *MTBC* species are nomenclatural validly published and *MTBC* taxonomy has been under constant scientific revision, with future changes being expected. Despite their genetically close relationship, they differ in epidemiology, pathogenicity, geographical range, host preference, and severity of tuberculosis disease in humans. Genetically, all members of this complex are highly conserved, possessing 99.9% similarity at the nucleotide level and identical 16S rRNA sequences (Peters et al., 2020). *M. tuberculosis* species, the most common pathogens in humans, can be further divided into genetic groups that also show differences in their levels of virulence, immunogenicity, and geographical distributions (Gagneux and Small, 2007). *M. bovis* BCG is one of the most common *MTBC* organisms found in clinical laboratory and is used to treat bladder cancer (DeGeorge et al., 2017; Larsen et al., 2020). *M. bovis* BCG is one of the most commonly administered vaccines and its complications include disseminated BCG disease which is rare but increasingly reported in immunodeficient children (Hesseling et al., 2007; Ritz et al., 2009). It is clinically important to differentiate *MTBC* members from other mycobacterium species considering the differences in resistance and treatment response rate to antibiotics (Scorpio and Zhang, 1996). The ability to distinguish between strict human and zoonotic tuberculosis and to trace source exposure during epidemiological studies is critical for the infection control process (Djelouadji et al., 2008).

Characterization of the proteome of *MTBC* is essential for differentiation at the species level with the proposed approach. It has been shown that during infection some expressed proteins vary during different stages of infection while others are present during the entire infection (Mehaffy et al., 2012). Some of the *Mtb* secreted proteins are essential for pathogenesis of the disease and can be potential targets for vaccine development (Brock et al., 2001). For example MtbHU (a small dimeric nucleoid-associated protein), HspX/14-kDa (α-crystalline homologue), the molecular chaperone GroEL2, and bacterioferritin (BfrB) are essential for growth and pathogenesis of *Mtb* and can serve as target for treatment (Bhowmick et al., 2014). Any delay in correct diagnosis and treatment may lead to sepsis and emergence of resistance to available drugs, this can be one of the driving forces for the search of new drugs that will take years or decades to develop. There are many known mechanisms of resistance such as possible mutations of the *pncA* and *rpoB* gene, hydrophobic and waxy cell wall composition, slow growth rate while dormant, capacity to suppress the host immune response, and an efficient efflux pump system (Scorpio and Zhang, 1996; Somoskovi et al., 2001; de Steenwinkel et al., 2010; Louw et al., 2011). Outside of these known resistance pathways there may be many unknown resistance mechanisms which need to be explored as well. Therefore, a rapid and accurate differentiation of closely related pathogens is crucial. However, most of the recent methods that are based on phenotypes, biochemical characteristics, nucleic acids, and molecular systems (Somoskovi et al., 2008) for *MTBC* species differentiation and identification have suboptimal diagnostic performance. In addition, next generation sequencing is laborious to perform (Hughes et al., 2012). Even MALDI-TOF, which has revolutionized the clinical microbiology identification of mycobacterium (Mather et al., 2014), could not discriminate *MTBC* species (particularly as IVD cleared test) (Chen et al., 2013; Lévesque et al., 2015).

In addition to the need of a more discerning assay, it is also essential to have a pre-analytical method to render the microbes non-viable. A unique incubation with pre-incubation solution (Thermo Fisher Scientific, proprietary) followed by rigorous mixing in buffered solution method was developed for inactivation of *MTBC* to overcome shortcomings of other inactivation methods such as heat treatment (Doig et al., 2002), exposure to lethal irradiation (Murdoch et al., 2012), multiple centrifugation and washing steps etc. In this study, we utilize micro (nano) flow liquid chromatography-mass spectrometry (LC-MS) to separate proteins from microbial extracts and analyze them in a high-resolution accurate mass (HRAM) orbitrap mass spectrometer. We used a workflow consisting of cell sonication, solid-phase extraction (SPE) purification, mass spectrometry, and computational algorithms to achieve our high identification accuracy for these organisms. The goal of the present study was to provide a proof-of-principle experiment employing orbitrap LC-MS for discrimination of mycobacterium species. This is the first study in which differentiation of the *MTBC* at a species level is achieved by employing a mass spectrometry driven approach with the potential to discriminate all types of closely related *Mtb* pathogens.

## Materials and Methods

### Strain Selection and Reference Identifications

*Mycobacterium* strains belonging to the species *M. africanum* (*syn. M. tuberculosis; Genetic lineage*), *M. tuberculosis*, *M. bovis*, *M. bovis* BCG and *M. microti* being of paramount clinical relevance were selected as representatives for the *Mycobacterium tuberculosis* complex). Although *M. canetti*, *M. caprae*, *M. orygis*, *M. suricattae*, *M. pinnipedii*, and *M. mungi* also form the *MTBC*, we excluded them in this study due occurrence as causative agents of rare zoonotic human TB infections and their partially unclarified taxonomic status. Key objective to this study was providing an initial proof-of-principle towards the diagnostic potential of our novel LC-MS assay only. Taxonomic anchors were defined by ATCC type material and selected for the subsequent analysis (*M. tuberculosis* ATCC 27294™, *M. microti* ATCC 19422™, *M. africanum* ATCC 25420™, and *M. bovis* ATCC 19210™ (Note: This type strain appears to be *M. bovis BCG* by ARUP internal PCR based validation). In addition, 37 recent US clinical isolates obtained by Associated Regional and University Pathologists (ARUP) laboratories (7 clinical isolate strains of *M africanum*, 10 *M. tuberculosis*, 10 *M. bovis* and 10 *M. bovis* BCG) were analyzed in order to assess assay robustness. ATCC type material was obtained pre-identified in accordance with ATCC identifications procedures; all other recent clinical isolates were identified by quantitative (real-time) PCR and meltcurve analysis with a multiplex probe set to obtain a reference identification (Pounder et al., 2010) prior to LC-MS data acquisition. All strains of recent clinical origin were collected throughout the year 2019 at ARUP laboratories, with no additional metadata being available (double blinded clinical identifiers).

### Chemicals and Reagents

Optima LC-MS grade Water (H_2_O), Optima LC-MS grade acetonitrile (ACN), and Optima LC-MS grade formic acid (FA) were purchased from Fisher Scientific (Fair Lawn, NJ).

### Sample Preparation

#### Preparation of Whole Cell Extracts (WCE) for *M. tuberculosis* Complex Species

ATCC strains and clinical isolates of *M. tuberculosis* complex were cultured on 7H11 agar plates for approximately 18-24 days in a CO2 incubator maintained at 37°C in a BSL3 laboratory. A 1 µL loopful of colonies was transferred into sonication vials (Thermo Fisher Scientific, proprietary). Subsequently the pre-incubation solution containing alcohol (Thermo Fisher Scientific, proprietary) was added to the cells at room temperature (RT, 20-25°C) and following a short centrifugation step (12,000 x g for 2 minutes at RT) the supernatant was discarded and then the pellet was suspended into 100 µL of incubation solution containing formic acid and acetonitrile (Thermo Fisher Scientific, proprietary). The cell lysate were incubated for 20 minutes (vortexed once at the 10-minute mark for 2 seconds), followed by sonication for one minute at 50% amplitude. Cells were diluted with 100 µL dilution buffer containing acetonitrile (Thermo Fisher Scientific, proprietary) and centrifuged for 5 minutes at 12,000 × g at RT. The supernatant was collected in low protein binding (LBE) Eppendorf tubes and stored at -80°C if not immediately used for LC-MS analysis.

#### RP4H SPE Protocol

The whole cell extract (WCE) was diluted with SPE buffer-1 (Thermo Fisher Scientific, proprietary) before Solid-Phase Extraction (SPE) cleanup. Reverse Phase monolith (RP4H) SPE tips (Thermo Fisher Scientific, proprietary) were placed in 96 well-plate and conditioned and equilibrated with 50 µL proprietary SPE buffer-2 (Thermo Fisher Scientific, proprietary), respectively. A centrifuge (Laboratory centrifuge 4-15C, Thermo Fisher, Osterode, Germany) capable of handling two 96 well-plates was used for centrifugation at 2000 × g for 2 minutes at RT. Diluted WCE (50 µL) was loaded into the SPE tips and centrifuged. The tips were washed with 50 µL of proprietary SPE buffer-3 and placed in liquid chromatography (modified Easy-1000, Thermo Fisher Scientific, proprietary) autosampler plate for further online elution and mass spectrometric analysis. Blanks were treated as negative controls and were prepared in a similar way as described above, instead of cell lysate water was added to the SPE tips.

### LC-MS Analysis

All experiments were performed on a micro flow Liquid chromatography-Mass spectrometry (LC-MS) system. The liquid chromatography (LC) system was connected to a Q Exactive™ HF Orbitrap™ mass spectrometer (Thermo Fisher Scientific, San Jose, CA). Mobile phase A consisted of 0.2% FA, 10% ACN in H2O and mobile phase B consisted of 0.2% FA in ACN. Elution was done using gradient elution where after flow stabilization at 4 µL/min for 2%B, percent of B was increased to 33% B in 5 min. The LC and mass spectrometer were controlled by Xcalibur software version 3.0 (Thermo Fisher Scientific). The mass spectrometer was operated in positive electrospray ionization (+ ESI) mode. The general mass spectrometric conditions were: The spray voltage was set at 2.0 kV. The capillary temperature was 325°C and S-lens RF level was set to 65. The maximum injection time was 200 millisecond and 40 microscans were used. Automatic Gain Control (AGC) target was 3e6, resolution was 120,000 for MS. Intact protein MS mode was used with trapping gas pressure set to 0.2 and C-trap charge detection was set OFF. Mass analysis of proteins was performed in the range from 450 to 2000 *m*/*z* for full scan mode.

### Data Processing and Species Identification

**Figure 1:**
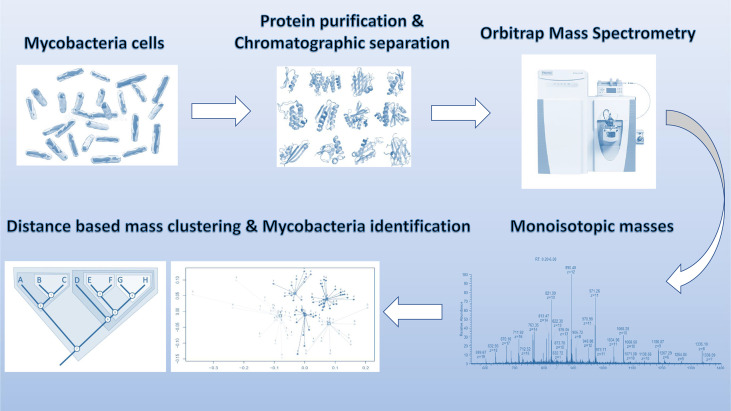
Schematic overview of sample processing, data acquisition and analysis steps employed in stepwise analysis of LC-MS Orbitrap data.

The raw data was deconvoluted (proprietary algorithm) in real time to monoisotopic masses (i.e. list of proteoforms). Subsequently, the data was processed with in-house algorithms for building of the database and identification of mycobacterium species. The individual data acquisition and processing steps are depicted in **Figure 1**. Each strain in the analysis was measured in two technical replicates for database construction and subsequent species prediction. Each ATCC strain in the analysis was also analyzed in six replicates (two technical replicates from three biological replicates) for the estimation of technical reproducibility of the sample processing and measurement.

Processing of the mass spectra was performed by Thermo Fisher proprietary software to deconvolute spectra in *m*/*z* space into monoisotopic protein masses between 5 and 40 kDa. Measured monoisotopic masses from each measurement were mass aligned to construct a list of consensus markers. One-way ANOVA was used to select top 200 consensus markers that have the most predictive value for the differentiation of the species. Species prediction was accomplished by a Thermo Fisher Scientific proprietary distance-based clustering algorithm. The robustness of species prediction was simulated by leave-one-out iterations where each sample was tested against a database constructed without data for the same strain (training and test dataset). The data will be made available on reasonable request.

## Results

### Inference and Determination of LC-MS Proteome Patterns

**Figure 2:**
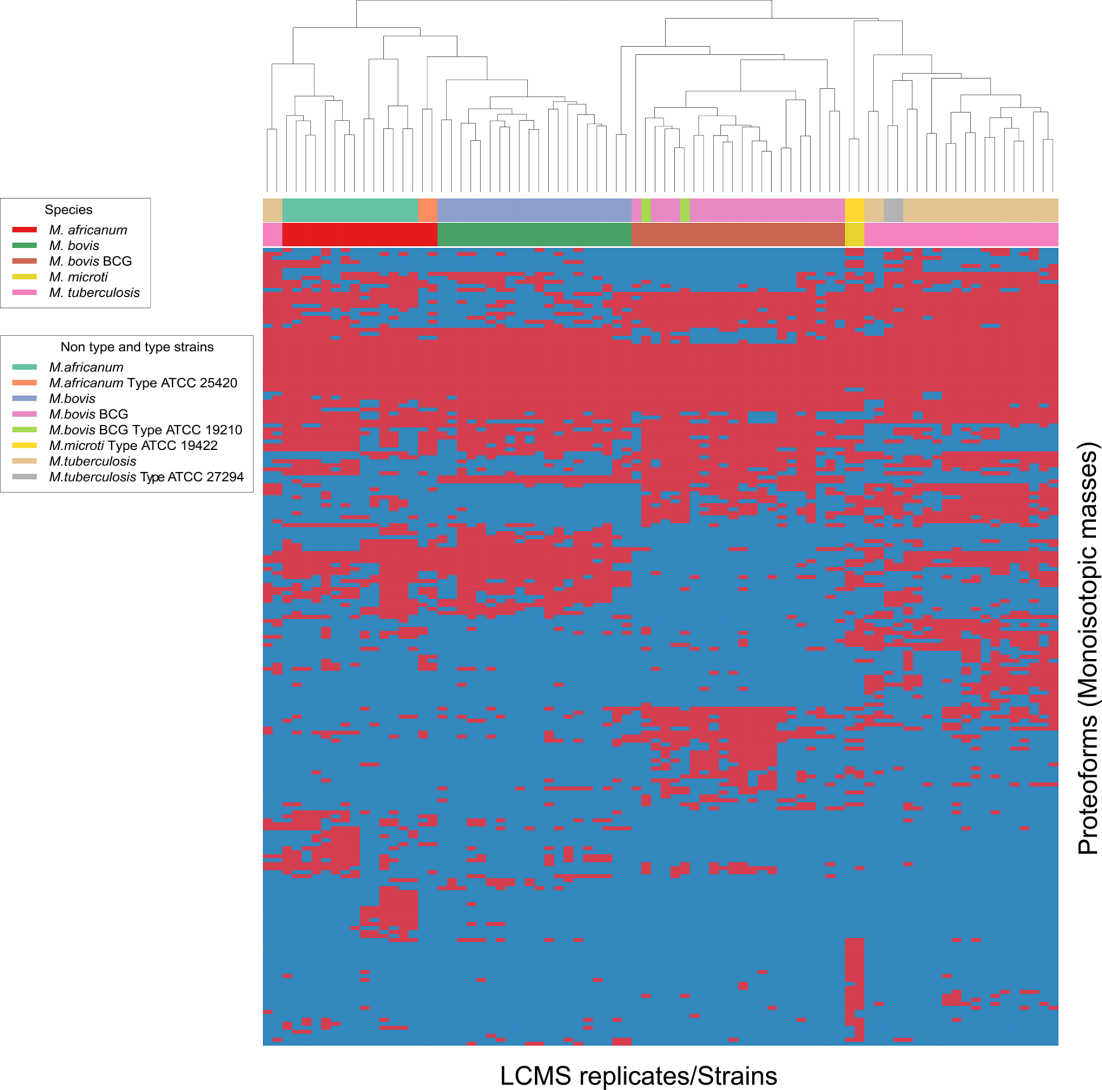
Heat map generated for all 4 species analyzed indicating top-200 discriminatory proteoforms. Horizontal bar on top, indicates the color assignment for the type and non-type strains used in the study, horizontal bar below indicates species assignment alone for visual clarity (see legend). Mass spectrometry data is here depicted as a heatmap, where rows represent individual monoisotopic masses (deconvoluted protein masses) and columns individual strains (technical LC-MS replicates for each strain were merged to a single leaf in the horizontal dendrogram) of the study cohort. Data has been converted to a binary state, where presents of a protein mass in each strain (species) is encoded as 1 (red) and absents 0 (blue). The representation indicates proteoform overlap and non-overlap between strains among all species due to their closeness at species level. Unique proteoforms detected among individual species specifically enable the classification down to species level (Specific number of exclusive red blocks for a given taxon). Central red block of proteoforms is shared between the species of interest and is non-species specific (conserved). The dendrogram shows that the *M. bovis* and *M. bovis* BCG clinical isolates are split in two separate branches, allowing discrimination of these fundamentally different strains (non-BCG and BCG strains). Additional work is required to understand proteomic diversity of atypical isolates, considering that a reliable identification must ideally capture all clinically relevant variants of a given species as paramount criterion to microbial diagnostics.

To verify whether different *MTBC* species show unique MS spectrum profiles, the masses of the deconvoluted proteoforms from each species were aligned to construct a list of consensus markers (clustering of monoisotopic masses). Subsequently, we could depict common (shared) proteoforms from deconvoluted spectrum profiles in order to determine visually common and non-common proteoforms (**Figure 2**). Unique masses per species were those masses present in all or most replicates of a given species and predominantly absent from replicates from other species. To verify whether different *MTBC* strains show similar mass spectral profiles, we analyzed 37 clinical isolates of *MTBC* with the depicted LC-MS analysis workflow (**Figure 1**). Consistent with the mass spectrum profiles obtained from the ATCC type strains, all clinical isolates exhibited a similar pattern (data not shown), which comprises common proteoforms ranging from 5 to 40 kDa. Accordingly, similar elution profiles and identified proteins, result in consistent species-specific proteome patterns.

### Differentiation of MTBC Species

We next evaluated whether the MS spectrum profile and common proteoforms obtained from different species within *MTBC* can differentiate the species within the complex. Based on the different expression of species-specific protein profiles (unique masses), we developed species-specific databases capable of rapidly differentiating *MTBC* at species level with very high identification accuracy.

As represented by the clustered monoisotopic masses which were visually depicted as heatmap in **Figure 2**, the unique protein signatures are successfully able to differentiate *M. bovis* and *M. bovis* BCG from other taxa of the *MTBC* in individual distinct monophyletic clusters. Similarly, *M. tuberculosis* and its genetic lineage syn. *M. africanum* as well *M. microti* are monophyletically differentiated based on unique protein profiles. The results of the mass clustering were in accordance with the alternative identification obtained by qPCR meltcurve analysis as described above under material and methods. However, a single *M. tuberculosis* isolates was falsely clustered among the *M. africanum* (sensu stricto) isolates. This misplaced isolate is obvious at the very left of the heatmap-depicted clustered monoisotopic masses (**Figure 2**). This result disagrees with our reference-based identification and can likely be explained by proteoforms which might be rarely expressed by atypical *M. tuberculosis* strains. This inconsistency will require further investigation with additional *M. tuberculosis* and *M. africanum* strains. Subsequently, the classification results returned from the training and test data definitions returned very high success rates for the clinically important species *M. tuberculosis* and *M. bovis* BCG, while *M. africanum* and *M. bovis* returned slightly inferior results (100% correct identification vs. 90% and 88% respectively). Reciprocally, identification success is lowered for *M. africanum*, by false hits against *M. bovis* but not *M. tuberculosis* and for *M. bovis* sensu stricto against *M. bovis BCG*. The results of the species prediction are given in **Table 1**. *M. microti* was excluded from the analysis due to representation of the type strain only (eg. training and test dataset as described under material and methods accordingly would be identical).

**Table 1:** Classification success for identification of clinically prevalent *MTBC* species.

Species	N	Identification success (Top match only)	Combined top 3 matches for each data file
*M. africanum*	16	88%	41 hits to *M. africanum*,
			7 hits to *M. bovis*
*M. bovis*	20	90%	56 hits to *M. bovis*
			4 hits to *M. bovis* BCG
*M. tuberculosis*	22	100%	64 hits to *M. tuberculosis*
			2 hits to *M. africanum*
*M. bovis BCG*	22	100%	66 hits to *M. bovis* BCG
**Total**	**80**	**95%**	

N refers to the total number of mass spectra measured for each species. Total identification success is presented as ‘percent success’ as a result to predict the target species with the employed algorithm. For each data file, the 3 closest matches were collected (additional and including the top match) if applicable, and the species results for these top 3 matches is displayed in the right-hand column. In all cases the best match was to another strain of the same species, but in some few cases the 2^nd^ or 3^rd^ best match was for a different species as detailed in the column on the right. In the analysis we used only the type-strain of the *M. microti*, accordingly no species predictions are returned in this case as training and test data would be identical.

Based on the different expression of species-specific protein patterns, we were capable to differentiate the clinically most prevailing species within the *Mycobacterium tuberculosis* complex down to species level. Differential proteoforms may serve as potential biomarkers for identifying these microorganisms in patient samples in additional confirmatory studies using clinical specimens. These results outline the potential to the employed LC-MS technology, as a first-line platform in the rapid and accurate identification of *MTBC* species in routine clinical microbiological laboratories.

## Discussion

In this study, we evaluated the resolution power of LC-MS as a novel method to delineate clinically relevant closely related *MTBC* species as a model system for challenging identifications. Rapid and accurate identification of microbial infections is essential for accurate patient supervision. Especially for *Mtb* infection, differentiation of *MTBC* species is very critical for the accurate disease diagnosis, starting drug therapy, to control the spread of disease, for public health surveillance and appropriate patient case management. *MTBC* infections have been one of the most important and common mycobacterial clinical pathogens, especially in immune deficient adults and pediatric patients. Among *MTBC* species, *Mycobacterium tuberculosis* is more predominant in clinical samples and environmental samples like water and soil which contributes almost exclusively to disseminated *MTBC* disease.

Acid-fast staining and microscopy as a routine technique was the most common and least expensive option for laboratory diagnosis of human tuberculosis. However, this technology has a low sensitivity and specificity and does not provide information on the identity of the pathogen. Comparatively rapid nucleic acid amplification test (NAAT) (Derese et al., 2012) and two-step PCR processes have been used for differentiation of *MTBC* species (Pinsky and Banaei, 2008; Pounder et al., 2010; Reddington et al., 2012; Nikam et al., 2013). However, these techniques fail to differentiate *MTBC* species, is more laborious and complexity of multiplex RT-PCR can be a problem. Routine laboratory culture, chemical testing procedures, and nested PCR targeting (Sinha et al., 2016) assays can differentiate *MTBC* members at the species level but the long identification process is not practical for rapid diagnosis of patients in need of immediate therapy. Alternatively, molecular methods such as GenoType MTBDRplus test (Hain Lifescience, Nehren, Germany) (Richter et al., 2004) or DNA probe assays (Kasai et al., 2000; Niemann et al., 2000) have been able to identify mycobacterial isolates as *MTBC* species, but lack capability to identify the isolate to the species level and limitation of it reliability on bacterial cultures to produce enough amount of bacterial DNA. Differentiation of *MTBC* species is possible based on intergenic spacers analysis in the genotyping of *M. tuberculosis* such as the exact tandem repeat D (ETR-D) (Frothingham and Meeker-O’Connell, 1998) and aliased mycobacterial interspersed repeat unit 04 (MIRU04) (Supply et al., 1997). And also, based on molecular methods such as the major polymorphism of tandem repeat (MPTR) sequencing (Frothingham, 1995), single nucleotide polymorphisms (SNP) in the *pncA* gene (Djelouadji et al., 2008), the *oxyR* locus (Sreevatsan et al., 1996), the detection of deleted regions (Somoskovi et al., 2008), the restriction fragment length polymorphism of the *hupB* gene (Prabhakar et al., 2004), *gyrB* gene based differentiation (Chimara et al., 2004; Arnold et al., 2005), and mycobacterial interspersed repetitive-unit-variable-number tandem-repeat (MIRU-VNTR) typing and spoligotyping (Godreuil et al., 2007). Previous methods could differentiate the *MTBC* at the species level but are often complicated by low sequence variability at the nucleotide level. However, these methods may have some limitations when encountered with rare species or atypical strain variants, and do not have the ability to differentiate all *MTBC* species such as *M. pinnipedii* and *M. microti* (Ayele et al., 2004). Such time-consuming and laborious methods hamper proper treatment of patients with respect to the different antibiotics and supportive treatments and are not practical for surveillance purposes. MALDI-TOF methods have been applied to mycobacterial (Mather et al., 2014) species identification and have shown to be a reliable technique in the routine laboratory. Although, MALDI-TOF for discrimination of mycobacterium is relatively rapid and reasonably accurate, however, this technique also could not differentiate *MTBC* species in clinical laboratories. Recently, a cheap and rapid new High Resolution Melting (HRM) assay for identification and differentiation of Mycobacterium tuberculosis complex samples was developed but it still had limitation to differentiate *M. bovis* from *M. bovis BCG* and *M. caprae* as well as *M. africanum* from *M. tuberculosis* (Landolt et al., 2019).

In contrast, in the present study, we demonstrated that ESI-Orbitrap LC-MS could be used for accurate identification of taxonomically complex microbial species. One of the advantages of using ESI is that it ionizes analytes to produce multiple charge states, bringing the mass to charge ratio of larger proteins into the window of the usual mass spectrometer range. These higher mass proteins show increased discriminatory power for speciation between highly similar species which may be a reason for the improved success of this approach above other Mass Spectrometry approaches. While in this study protein identification was not carried out, the setup would allow the further characterization of the discriminatory proteins through a top-down proteomics workflow. These protein identifications if characterized as related to resistance or virulence could also have possible applications in routine clinical diagnostics (LeDuc et al., 2004). While novel diagnostic approaches employing complex LCMS technology might not be directly suitable and useful in developing countries, great benefit arises to many other countries and reference laboratories which will have access and funds to high resolution MS systems where the incidence of TB infection is still high.

ESI-LC-MS has been applied to study secretome, cell wall proteome, membrane proteome, and PTM profiling of *M. tuberculosis* (Ge et al., 2003; Gunawardena et al., 2013). However, no study has demonstrated the applicability of ESI-Orbitrap LC-MS in mycobacterial identification. Also, Dukik et al. demonstrated the use of ultra-high-resolution mass spectrometry technique for identification of closely related dermatophytes but no other report yet outlined this technique for microbial identification (Dukik et al., 2018). To the best of our knowledge, this is first study showing applicability of this technique for *MTBC* identification and discrimination at species level with very high accuracy in a very short time utilizing unique masses (protein profiles) from each species. Our present study was optimized to have an LC-MS analysis method of 5 minutes and demonstrates its potential for rapid diagnosis which could impact treatment times. This method is universally applicable and can be beneficial in the veterinary and other health care related applications for fast and precise testing. The methodology could serve as a tool in the differentiation of *MTBC* members and detection of transmission in captive and other animal populations. The *MTBC* consists of the closely related organisms *M. tuberculosis, M. africanum, M. bovis, M. bovis BCG*, and animal born zoonotic species rarely identified in patients. The accurate molecular species identification within the *MTBC* is paramount to guide public health and primary care decisions more effectively due to species specific epidemiology, partial specificity in their host spectra (e.g. zoonotic taxa), geographic prevalence and drug susceptibility. *M. tuberculosis* and *M. bovis* may highly differ in contact tracing. Treatment with pyrazinamide can be excluded in case of *M. bovis* or *M. bovis* BCG as they are naturally resistant to the drug. *Mycobacterium bovis* Calmette-Guerin (BCG) is a live, attenuated strain of *M. bovis*. It is widely used as a vaccine against tuberculosis worldwide, and BCG is very effective for the treatment of transitional cell carcinoma of the bladder. Typically, it may require months to years for physicians to identify patients being infected with BCG disease upon exposure. Also, *M. bovis* mostly causes infection in cattle, deer and other mammals but the consumption of unpasteurized infected cow milk or transmission from infectious tuberculosis patient harboring *M. bovis* can cause disseminated infection in humans. The distribution of the various species is as follows and may vary according to the patient population served: *M. tuberculosis* (95%), *M. bovis* (2%), *M. bovis BCG* (1.5%), and others (1.5%) (ARUP internal communication Mycobacteriology department; Incident rates 2020).

In this study, we show potential for utilizing LC-MS and algorithmic discrimination at the species level for *MTBC* species with high classification success for the clinically most relevant entities. This method offers the ability to generate classification models from large numbers of spectra in a relatively rapid and flexible way. The aim is to determine a common signature among spectra for each of the identified taxa in such a way that spectra of test isolates can be classified accurately. The utilized algorithm uses proteoforms in the approximate mass range of 5 to 40 kDa which were obtained from the mass spectra. A characteristic protein profile in this range provides enough information for the differentiation of various clinically relevant mycobacterial species and clinical isolates at least at species level with accuracy as high as 100% as shown in **Table 1**. While molecular taxonomy of mycobacterium is in general very dynamic, and species and subspecies (lineages) boundaries are evolving with advancement of laboratory technologies, our approach demonstrates the possibility to identify strains to their species by mass spectrometry in a method that matches their current genomic reference.

## Conclusion

Whole-protein top-down LC-MS analysis has significant diagnostic potential because of its ability to detect proteins routinely in a wider mass range and the method flexibility which can be optimized to provide the necessary protein coverage, e.g. if needed even to discriminate below the species level i.e. at the lineage or strain level. The accurate determination of protein masses, separation of high numbers of individual proteins, and high-resolution enabling detection of single-amino-acid substitutions are responsible for this high performance. Our algorithms showed a potential to recognize individual strains that could be applied in epidemics or outbreak scenarios. This novel assay is the first to use LC-ESI-MS to accurately identify the *M. tuberculosis* complex to the species level. The assay can rapidly, precisely, and accurately discriminate *M. tuberculosis* complex. Most importantly it can discriminate among *M. tuberculosis* complex isolates to the species level. These species are closely related members of the *M. tuberculosis* complex but very diverse pathogenetically. Having an effective and accurate way to distinguish these taxa will help in understanding the epidemiological behavior of these pathogens with a goal of supporting the development of improved diagnostics and treatment. However, our analysis was limited to 7H11 agar medium only and not yet tested with liquid culture medium as well as Loewenstein-Jensen agar which need to be assessed in the future. Additional research is required in targeting a wider species and strain panel, including the entire *MTBC* and its rarer zoonotic taxa, a large sample size and fine-tuned protocols. Algorithmic improvements to increase and further test classification success and to provide confirmatory experimental data to this first proof of principle study splitting clinically common species in the *M. tuberculosis* species complex will be required to underline the high potential of Orbitrap LCMS technology.

## Data Availability Statement

The raw data supporting the conclusions of this article will be made available by the authors, without undue reservation.

## Author Contributions

AOB and SS have drafted the work, AOB, SS, JF, JBS, and APB designed research, AOB performed experimental work, data acquisition, analysis, AOB and JBS conducted review and editing, JK and JBS performed interpretation of data and computational analysis used for the work, and JF and APB provided funding acquisition, project administration, and resources. All authors contributed to the article and approved the submitted version.

## Conflict of Interest

Authors JF, JBS, and JK were employed by company Thermo Fisher Scientific.

The authors declare that this study received funding from Thermo Fisher Scientific. The funder was involved (equal partner) in the study design, assisting with providing tools and assistance in analyzing the data, and technical interpretation of results. The funder was not further involved in the collection, interpretation of data, the writing of this article (beyond assisting with technical details for the materials and methods and textual review) or the decision to submit it for publication.
